# Quantification of Wear and Deformation in Different Configurations of Polyethylene Acetabular Cups Using Micro X-ray Computed Tomography

**DOI:** 10.3390/ma10030259

**Published:** 2017-03-03

**Authors:** Saverio Affatato, Filippo Zanini, Simone Carmignato

**Affiliations:** 1 Laboratorio di Tecnologia Medica, Istituto Ortopedico Rizzoli, Via di Barbiano 1/10, 40136 Bologna, Italy; 2 Department of Management and Engineering, University of Padova, 36100 Vicenza, Italy; filippo.zanini@unipd.it (F.Z.); simone.carmignato@unipd.it (S.C.)

**Keywords:** cross-linked PE, vitamin-E doped PE, standard PE, hip simulator, μCT, daily activity

## Abstract

Wear is currently quantified as mass loss of the bearing materials measured using gravimetric methods. However, this method does not provide other information, such as volumetric loss or surface deviation. In this work, we validated a technique to quantify polyethylene wear in three different batches of ultrahigh-molecular-polyethylene acetabular cups used for hip implants using nondestructive microcomputed tomography. Three different configurations of polyethylene acetabular cups, previously tested under the ISO 14242 parameters, were tested on a hip simulator for an additional 2 million cycles using a modified ISO 14242 load waveform. In this context, a new approach was proposed in order to simulate, on a hip joint simulator, high-demand activities. In addition, the effects of these activities were analyzed in terms of wear and deformations of those polyethylenes by means of gravimetric method and micro X-ray computed tomography. In particular, while the gravimetric method was used for weight loss assessment, microcomputed tomography allowed for acquisition of additional quantitative information about the evolution of local wear and deformation through three-dimensional surface deviation maps for the entire cups’ surface. Experimental results showed that the wear and deformation behavior of these materials change according to different mechanical simulations.

## 1. Introduction

Material selection and component design in total hip replacement (THR) are important factors in the performance and durability of total joint replacements [1,2]. Unfortunately, wear of hip bearings exists and is a significant clinical problem. Controlled wear testing of total hip replacements in hip joint simulators is a well-established and powerful method, giving an extensive prediction of the long-term clinical performance [3,4,5]. Wear volumes measurement and geometrical characterization of worn bearing surfaces are fundamental for understanding wear mechanisms and improving technological solutions provided by manufacturers of prosthetic joint components [6,7,8]. One of the most important aspects of wear testing is the simulation of the actual wear conditions that occur within the human body once the joint is replaced with an artificial one. The current ISO 14242 defines loads, motions, and methods used for in vitro simulations [9]; these international guidelines identify three degrees of freedom (abduction/adduction, inward/outward rotation, and flexion/extension) and axial load. Nowadays, despite the overall success of hip joint simulators in predicting in vivo behavior of a wide variety of materials and bearing combinations, constant evaluation and refinement of the test protocols are of paramount importance. Several attempts have been made to simulate more aggressive in vivo conditions—for example, by adding third body wear particles during the test [10,11,12] or artificially aging of ultrahigh-molecular-weight polyethylene (UHMWPE) acetabular cups [13]—with the aim of replicating some of the adverse conditions that may occur in the artificial joint. However, these more aggressive wear protocols have yet to be universally accepted or standardized. Experimental hip wear simulators have been developed in order to enhance the existing knowledge and to assess the effect of various parameters on UHMWPE wear mechanisms, but still only modest information is available on in vivo loads and kinematics responsible for the wear of the polyethylene (PE) inserts. The ISO 14242 standard, despite being revisited over time, is based on analytical studies dating back to more than 40 years ago [14,15] and gives indication only about level walking. However, THR components—during the day and throughout the human life—experience a much larger variety of more demanding physical activities, which imply complex combinations of load and motion. Currently, implanted patients’ request complete mobility after surgery, requiring various loading scenarios (stair climbing, chair sitting, squatting) that the implant must allow, even a short time after implantation. Thus, in vitro wear simulation of several and (possibly) more demanding daily physical activities different from level walking are needed, and could provide important information still missing in the knowledge of the tribological aspects and wear phenomena, which lead to THR failure. This field of research is the last frontier about the wear assessment of orthopedic devices. In fact, some scientists think that the wear tests should reproduce realistic simulations of various motor tasks of daily living (chair sitting and rising, squat, stair ascent, etc.). Affatato and coworkers, in previous studies about the knee simulation [16,17,18], found that the kinematic and load data applied to a knee simulator showed an accurate reproduction of the various motion and load patterns. To the authors’ knowledge, no existing literature/reports are available in regards to this field, applied to the hip simulation, and no tests have been carried out on a hip wear simulator. The so-called “patient’s daily activities” have to be considered as a worst-case scenario for testing, as well as for implant design. To cover these lacunae, based on previous studies [16,19,20], these experiences were translated to the hip simulator.

With this in mind, in a previous work [3], the wear behavior of three different polyethylene configurations was analyzed after performing an in vitro test under the simulating conditions recommended by ISO 14242. Following the first research, the wear test was chosen to be extended for an additional 2 million cycles using this modified ISO 14242 waveform in order to evaluate the wear effects due to a higher load. In particular, in this context, a new approach was proposed in order to simulate, on a hip joint simulator, the activities that represent the realistic simulations of various motor tasks of daily living (chair sitting and rising, squat, stair ascent, etc.), as better explained in our previous work [16]. The effect that these activities have on different polyethylenes’ acetabular cups (vitamin E-stabilized cross-linked PE, cross-linked PE, and standard PE) was analyzed in terms of wear and deformations by means of gravimetric method and micro X-ray computed tomography (μCT). The gravimetric method, which is the most commonly adopted method, provides only a global evaluation of weight loss due to wear. This outcome cannot be sufficient for an overall assessment of a dynamics wear test that also includes local distribution of wear over the worn surface and possible deformations [21]. Wear distribution and deformations could change depending on the load configuration and positioning adopted during the wear test and on material properties. Since μCT allows obtaining a holistic three-dimensional volumetric model of the scanned component with high-density surface digitalization in a relatively short time [22], it was employed in this work for a more complete evaluation of the tested components. Moreover, CT is particularly suited for analysis of polymeric components in comparison with contact coordinate measuring machines (CMMs). CMMs, in fact, may cause damage or unwanted deformations to the measured component due to clamping and probing forces.

## 2. Materials and Methods

### 2.1. Materials

Three different configurations of commercial polyethylene acetabular cups (32 mm inner × 50 mm outer dimensions) were coupled with 32 mm cobalt–chromium–molybdenum (CoCrMo) femoral heads. Three specimens for each polyethylene configuration were used for the test, and one other was taken as a control-check in order to estimate the total change in mass due to lubricant absorption, following ISO 14242-2. The tested specimens had been previously subjected to an in vitro wear simulation for 2 million cycles (Mc). Major details are available in literature [3]. After that first test, all the components were tested on the same hip simulator for another 2 Mc (for a total of 4 Mc).

### 2.2. Kinematics and Load Waveforms

**Figure 1:**
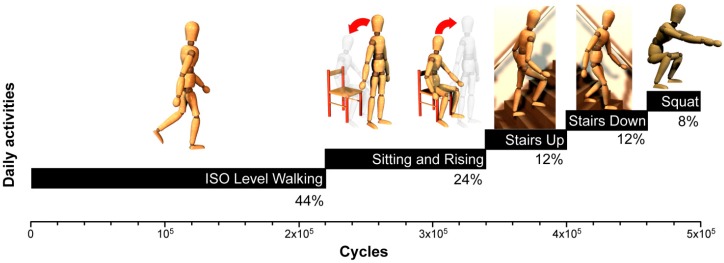
Distribution of high-demand daily activities during wear test.

The second in vitro wear test on the 12 specimens previously tested in [3] was performed for the other 2 Mc using a 12-station hip joint simulator (IORSynthe, Bologna, Italy). This wear test was performed on the same aforementioned specimens, using the same simulator, different kinematics, and same lubricating conditions used in [3]. In the previous study, a simplified gait cycle was reproduced, according to ISO 14242-3 and on the basis of a consolidated internal protocol [1,4,23]. Instead, a new approach has been introduced in this study with regards to the applied new kinematics. In fact, to make the hip wear simulation affordable, only four main typical activities of the normal patients’ daily life were considered: level walking, stair climbing and stair descending, chair sitting and rising, and deep squatting. Level walking is simulated according to current ISO 14242. The axial load data sets for the considered high-demand daily activities were derived from literature data [24]. In order to replicate a *worst-case scenario*, load parameters are in reference to a subject with a body weight of 1000 N. The distribution of the activities for the introduced hip wear test protocol has been established on the basis of several studies which monitored the everyday life of patients after total joint replacement [25,26,27]. These load waveforms were then modified in order to set the frequency of each motor task and to obtain a new relevant protocol. Figure 1 shows the various tasks applied during this wear test. The resulting protocol consisted of 44% level walking, 24% sit-and-rise activity, 12% stair climbing, 12% stair descending, and 8% deep squat.

Table 1 shows the axial load ranges for each motor task according to Bergmann et al. [24] and the rotation frequency imposed during simulation according to literature data analysis [25,26,27].

**Figure 2:**
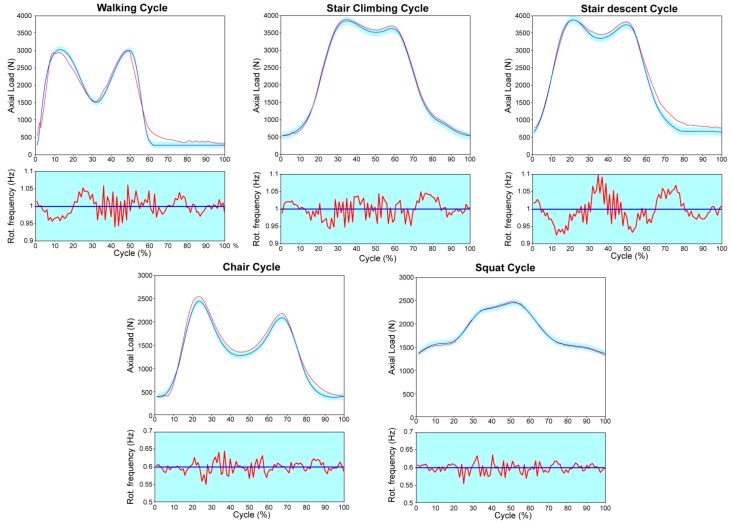
Load waveforms and rotation frequency for each considered motor task and machine response.

Input load waveforms for the considered motor tasks are shown in Figure 2.

### 2.3. Wear Test Details and Weight Loss Evaluation

Polyethylene acetabular cups (standard PE (ST_PE), cross-linked PE (XLPE), and cross-linked PE plus vitamin E (XLPE_VE)) were tested for 2 million cycles (Mc). The lubricant used was 25% (*v/v*) newborn calf serum balanced with distilled water, with 0.2% sodium azide (in order to retard bacterial growth), and 20 mM EDTA (ethylenediaminetetraacetic acid) to minimize precipitation of calcium phosphate. The weight loss of the cups was determined every 0.4 million cycles using a microbalance Cubis MSE 225 s-000-du (Sartorius, Goettingën, Germany) with a precision of 0.01 mg. Wear trend was determined from the weight loss of each acetabular cup, corrected by acetabular soak control; the wear rates, calculated from the steady-state slopes of the weight loss versus number of cycles lines, were obtained using least squares linear regression. The weight loss data were analyzed using a nonparametric Kruskal–Wallis (K–W) test and a least significance difference (LSD) as post hoc test. Statistical significance was set at *p* 0.05.

### 2.4. μCT Wear and Deformation Assessment

In a previous work of the authors [21], CT volumetric wear evaluation of wear-tested hip components was found to be well correlated to the weight loss assessed using gravimetric methods. In this paper, μCT potentialities were exploited in order to obtain additional information on how wear and deformation occur over the entire cup surface due to wear tests.

The CT measurement procedure consists of three main steps: (a) acquisition of bidimensional X-ray projection images of an object at several angular positions; (b) reconstruction of a three-dimensional model of the object; and (c) surface determination. A metrological μCT system was used, featuring a 225 kV microfocus X-ray tube, 2000 × 2000 pixels flat-panel detector (16 bit), temperature-controlled cabinet, and maximum permissible error (MPE) for length measurements equal to (9 + L/50) μm (where L is the length in mm) [28]. The use of a metrological CT system is necessary to obtain accurate and reliable measurement results. However, CT accuracy is highly dependent also on the user experience [29]. In particular, several acquisition parameters need to be properly selected. The spatial resolution of a CT scan is mainly influenced by several factors, including the size of the focal spot emitting the radiation and the voxel size (where voxel means volumetric pixel). The nine acetabular cups under test were CT-scanned with an electron beam with a power of 9 watts out of the possible 225 watts, allowing a small focal spot to be obtained. The voxel size, which decreases with the increase of the geometrical magnification of the object, was equal to 31 μm in order to fit the object to the detector field of view. CT-scanning parameters were optimized by comparing the effect of different setups on signal-to-noise ratio and on contrast of X-ray projections. The optimized parameters used for CT acquisition are listed in Table 2.

**Figure 3:**
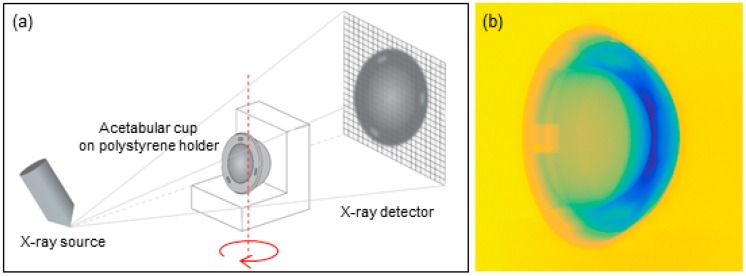
(**a**) Acetabular cup as it was positioned during the CT acquisition; (**b**) example of 2D projection image acquired by CT (RGB coded), showing that the polystyrene stand is not visible and does not disturb the measurement.

The sample positioning (see Figure 3a) was studied in order to minimize the Feldkamp effect, typical of cone-beam CT systems, and to localize the resulting artifacts over regions not affected by wear, as suggested in [6]. The mounting stand was made by polystyrene, since it has low X-ray absorption and does not compromise the measurement. In Figure 3b, a color-coded bidimensional projection of a cup is shown: it can be observed that the polystyrene stand is not visible.

Since the acetabular cups were retained in an appropriate liquid, they were carefully cleaned, dried, and allowed to stabilize inside the CT cabinet for 100 min before each CT scan. Stabilization was also required for thermal reasons and for avoiding unwanted movement of the object during the CT scan. After the X-ray 2D projections were obtained, 3D voxel models of the investigated components were reconstructed by means of a filtered back-projection algorithm. The software VGStudio MAX (Volume Graphics GmbH, Heidelberg, Germany) was then used for a direct elaboration of CT volumetric voxel data, with no need for a prior extraction of surface models (e.g., STL format) that could influence the analysis accuracy. As a first step, a local adaptive thresholding method [22] was applied to determine the surface location of the reconstructed model (i.e., definition of material boundary) with subvoxel accuracy by trilinear interpolation. In order to evaluate wear and deformations, each cup was scanned three times: (i) before wear tests; (ii) after wear test under ISO 14242; and (iii) after wear test under daily activities. The three reconstructed volumes were then aligned in VGStudio MAX to obtain deviation maps (see Section 3). Reliable maps can be generated only through an accurate alignment procedure. In this study, a two-step procedure was applied:

(1) Preliminary rough alignment: registration of each single component according to a primary, a secondary, and a tertiary datum reference. For each step, a geometrical element was defined and the axis/origin coordinate specified by that geometrical element was chosen.
(2) Fine adjustment of the previous alignment based on the current position of the objects.

**Figure 4:**
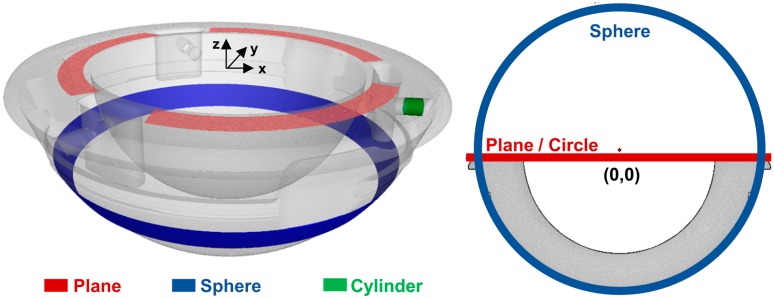
Unworn and nondeformed regions selected to create the geometrical elements used for the alignment (**left**) and 2D representation of the resulting fitting sphere, fitting plane, and intersection circle (**right**).

To perform the rough alignment (1), four geometrical elements were created by least-square fitting on unworn and non-deformed regions (see Figure 4) and were associated to specific axis/origin coordinates: (i) top plane obtained from points fitted on the red region (to define the x–y plane); (ii) sphere from points fitted on the blue region; (iii) circle created as intersection between plane and sphere (to define the origin of the coordinate system); and (iv) cylinder from points fitted on the green region (to define the x-axis).

The subsequent fine adjustment (2) was performed through a best-fit refinement constrained to the preliminary alignment, as allowed by the software VGStudio MAX. Using the same software, comparisons between components scanned before and after the two wear tests were conducted in order to achieve a detailed evaluation of detected deviations (see Section 3).

## 3. Results

**Figure 5:**
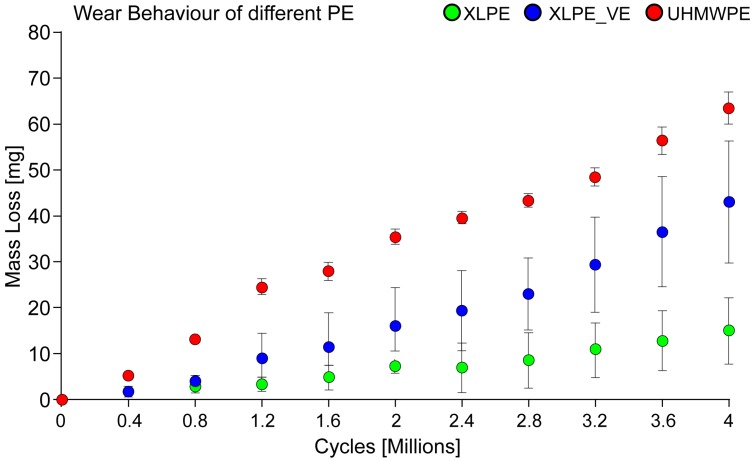
Wear trend of all different configurations of polyethylene (PE). XLPE: cross-linked PE; XLPE_VE: cross-linked PE plus vitamin E; UHMWPE: ultrahigh molecular weight PE.

Figure 5 shows the trend of the total mass loss (from 0 to 4 million cycles) evaluated using the gravimetric method during the two wear tests for the three sets of different acetabular cups.

As can be easily seen, in both the tests the mass loss decreased along the series STD_PE XLPE_VE XLPE. The XLPE acetabular cups maintained the lowest wear behavior through the wear test. From this picture, in which the mass loss is shown as a function of the number of cycles, it is interesting to note that the XLPE and XLPE_VE maintained the same wear behavior from 0 to 0.8 Mc. From 0.8 Mc to the end of the study, the XLPE_VE configuration wore more than the XLPE. On the contrary, the STD_PE configuration has shown an increase in mass loss more than 2 times than the other two configurations. At 4 Mc, significant statistical differences (*p* = 0.027) were observed between all the different PE configurations (Table 3).

In particular, with the LSD post hoc test, the statistical significance is observed between the two configurations STD_PE and XLPE. The slope values of the linear regressions representing the two tests (i.e., the wear rates) allowed us to gain more insights into the wear trends. In fact, it is interesting to note that the acetabular cups showed a different wear behavior in the two tests: with respect to the first test (from 0 to 2 Mc), all the samples underwent an increase in the mass loss, which was generally even higher in the second test (from 2 to 4 Mc). 

**Figure 6:**
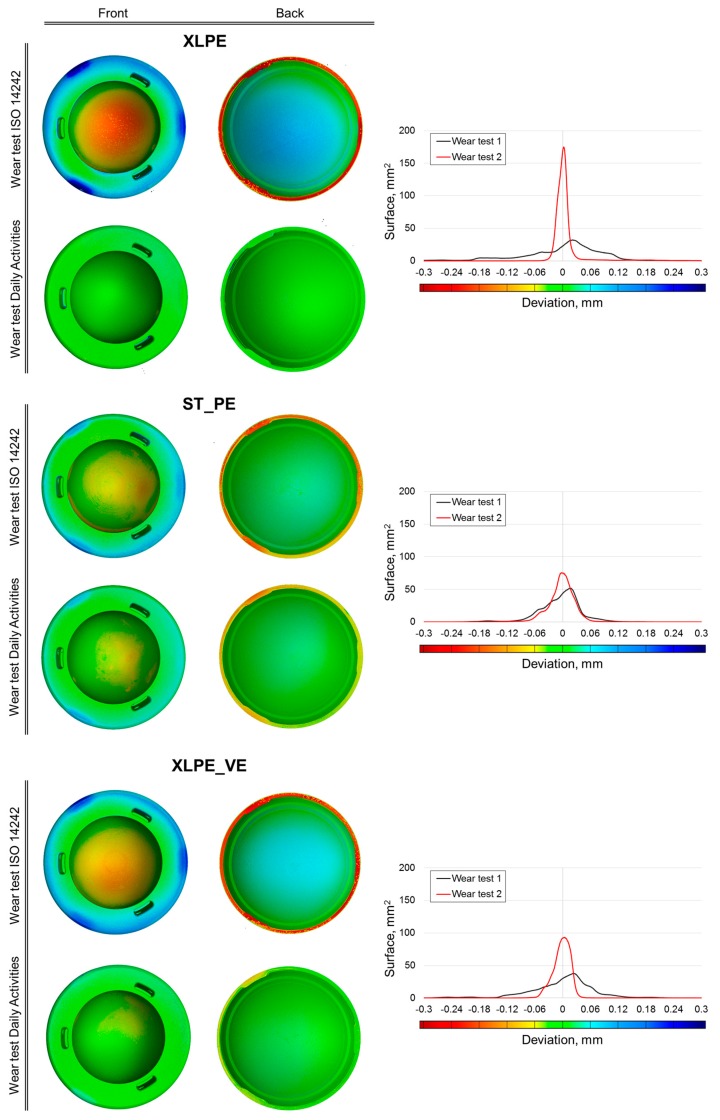
Comparison of deviation maps resulting from wear test under ISO 14242 and modified wear test under daily activities (one acetabular cup for each material). Histograms of the calculated deviations versus the entire analyzed surface of the selected object are shown.

Figure 6 compares the false-color-coded deviation maps obtained from μCT data on three representative acetabular cups (one example for each material type), where every color corresponds to a specific surface deviation (in millimeters) caused by the wear tests. In particular, “wear test ISO 14242” maps compare the as-produced cups with the same cups after the first wear test, while “wear test Daily Activities” maps compare the cups as they were after the first wear test with the same cups after the second wear test.

The range of the color scale was set between −0.3 and 0.3 mm to include the maximum deviations. Histograms of the calculated deviations versus the entire analyzed surface of the selected object are shown. Positive deviations correspond to areas where the deformations of the component caused a raising of the material surface position, while negative deviations correspond to areas that were lowered by deformations or wear. Results obtained with the two wear tests are both illustrated considering the front side and the backside of components.

## 4. Discussion

Numerous techniques have been developed to assess PE wear of hip components during simulator tests [30,31,32,33,34,35]. Recently, the use of μCT has become widespread in the wear assessment of knee tibial liner [36]. In addition, hip simulator studies have indicated that cross-linking can reduce wear that occurs in acetabular components by 95% [37,38,39]. Our specific objective was to propose a new approach in order to simulate, on a hip joint simulator, the activities of high-demand activities (HDA). The effect that these activities have on different polyethylenes’ acetabular cups (vitamin E-stabilized XLPE, XLPE, and standard PE) was analyzed in terms of wear and deformations by means of gravimetric method and μCT. Weight loss results obtained with the gravimetric method clearly showed an increased wear behavior with respect to our previous test. Moreover, both wear tests (the one under ISO 14242 and the one focused on daily activities) resulted in a decreasing mass loss along the series STD_PE XLPE_VE XLPE. It is stressed that the XLPE acetabular cups maintained the lowest wear behavior through the first and the last wear test, even if it was affected by more deformation than the other two configurations. The XLPE_VE acetabular cups, under HDA waveforms, showed an increasing trend for mass loss: more than 3 times with respect to the other two configurations (Table 2). μ-CT investigations showed that different wear and deformations can be achieved when different materials and/or different types of wear tests are considered, proving that a mere weight loss assessment is not sufficient for a complete understanding of components’ behavior. For example, even if XLPE’s cups should be more creep-resistant than UHMWPE’s, they were unexpectedly found to be most affected by deformations in the wear test under ISO 14242, followed by XLPE_VE’s cups. In particular, a significant deformation can be observed on the backside (blue surface in Figure 6), which is associated to a specular deformation on the opposite surface (internal calotte). For this reason, deviations registered on the internal calotte (red surface) can be attributed only partially to wear. Furthermore, considering the wear test focused on daily activities, deformations are very much reduced. The outcome is slightly different for the ST_PE’s cups: deformations due to the ISO 14242 wear test are less pronounced than for the other materials and are almost equal to deformations arising from the daily activity test.

To the authors’ knowledge, this is the first report on this matter that used the μCT to assess the evolution of local wear and deformations of acetabular hip components under HDA. Jedenmalm et al. [40] evaluated the “accuracy and repeatability of a 3D method for polyethylene acetabular cup wear measurements using computed tomography (CT) and found that the method could be used for in vivo wear assessment in acetabular cups”. Some other reports that used the μCT technique to quantify the volumetric loss are available on the knee components. Teeter and coworkers [29] validated a technique to quantify polyethylene volumetric wear in tibial inserts using μCT, and they observed no difference between μCT and gravimetric volume measurements, considering wear volumes higher than 32 mm^3^. In agreement, Engh et al. [41] used a μCT technique to quantify the polyethylene wear on retrieved knee components, and they found that this technique can determine the volume and location of wear of retrieved tibial liners. Recently, Parrilli and coworkers used a μCT technique to assess the volume loss of a femoral head and found that this “new protocol could be considered an important tool for wear assessment” [42]. In a recent work, Affatato et al. [21] found a good correlation between μCT and gravimetric volumetric wear measurements performed on polymeric hip inserts, with wear volumes ranging from 6 to 25 mm^3^. With this in mind, we wanted to approach a new method to assess wear and adding specific activities that would eventually stress the prosthesis to its limits. It is thought that high loads, together with low frequency, cause different stress conditions on the polyethylene liner; to better quantify these differences, the use of a μCT technique could help the researchers to improve material designs and simulation setups. Future research will address extending the knowledge of the tribological repercussion of the THR sizing as well as the applied load and their concurrent action, with the final aim of providing—to the surgeons and manufacturers—the indication of this combined effect.

The present study has a number of limitations. First, the data collected for motor tasks were taken from literature and not from patients. For this study, it was hypothesized that more demanding physical activities would more coherently represent the kinematics and loads experienced at the replaced hip during daily living. An evolution of this investigation would imply the combination of the motor tasks presented in this study also with the standard ISO level-walking protocol, for the possible final arrangement of a single global wear protocol meant to simulate an entire typical day of living, also according to the analyses of daily activities. Finally, a comparison between these in vitro results with explanted hip prostheses could help the surgeons. Further tests should be performed on this matter because, to our knowledge, no previous studies have been reported on a similar method, so a direct comparison is not possible. This is probably due to the fact that, in classic knee wear tests, the ISO standard imposes a simplified gait, whereas a real simulation should take into account the real patient’s conditions and, in particular, the activity level of its host.

Moreover, concerning the μCT analyses, further investigations are needed to assess the influence of the alignment operations. In fact, even if the alignment was performed considering only the unworn and nondeformed regions, this selection could be problematic, especially when analyzing highly deformed components. 

## 5. Conclusions

This paper summarizes the use of hip simulators used in the research of new hip joint materials. Controlled wear machines should routinely be used to qualify materials and elucidate wear mechanisms. Simulator tests can be used to conduct accelerated protocols that replicate particular *worst-case scenarios*, thereby establishing the limits of performance of materials. Experimental results showed that different configurations of polyethylene are characterized by dissimilar wear and deformation behaviors when different wear mechanisms are applied. In particular, both wear tests led to a decreasing mass loss along the series STD_PE XLPE_VE XLPE, with increased wear behavior obtained with the new proposed wear test focused on daily activities. In addition, under the ISO 14242 load profile, XLPE’s cups were determined to be, unexpectedly, the most affected by deformations, followed by XLPE_VE’s cups. On the contrary, wear tests focused on daily activities emphasize reduced deformations. The CT-based method presented in this paper is suggested by the authors as an effective way to determine the location of wear and deformations and analyze how deviation maps of the analyzed specimens can change by the effect of different loads and kinematics applied. Further investigations are planned to optimize the new wear test proposed in this paper, as well as to evaluate the uncertainty of μCT evaluations.

## Figures and Tables

**Table 1:** Axial load ranges for each motor task and the rotation frequency imposed during simulation.

Activity	Min Load (N)	Max Load (N)	Frequency (Hz)
Level walking	ISO 14242-3	ISO 14242-3	1.1
Chair sitting and rising	351.20	2776.4	0.7
Stair climbing	546	3951.3	1.0
Stair descending	611.7	4082.2	1.0
Deep squatting	1339.1	2505.8	0.7

**Table 2:** Computed tomography (CT)-scanning parameters optimized for the standard polyethylene (STD_PE) acetabular cups.

Parameter	Value
Voltage	194 kV
Current	46 μA
Exposure time	1415 ms
Projections	1500
Scanning time	35 min
Voxel size	31 μm
Physical filtering	No

**Table 3:** Cumulative mass loss (±standard deviation) for the three sets of PE acetabular cups tested. The *p*-values were obtained using a Kruskal–Wallis nonparametric test.

Cycles (Mc)	Mean (mg) ± Standard Deviation			K–W Test (*p*-Value)	LSD Post Hoc Test (*p*-Value)		
	XLPE	XLPE_VE	STD_PE		XLPE vs. XLPE_VE	XLPE vs. STD_PE	XLPE_VE vs. STD_PE
2	6.5 ± 4.0	16.1 ± 8.2	35.4 ± 0.6	0.039	NS	0.034	NS
2.4	6.9 ± 5.3	19.3 ± 8.7	39.6 ± 0.3	0.031	NS	0.026	NS
2.8	8.6 ± 6.0	23.0 ± 7.9	43.3 ± 1.5	0.027	NS	0.022	NS
3.2	10.8 ± 6.0	29.3 ± 10.4	48.4 ± 1.9	0.027	NS	0.022	NS
3.6	12.9 ± 6.5	36.5 ± 12.0	56.3 ± 2.9	0.027	NS	0.022	NS
4	15.0 ± 7.1	43.0 ± 13.3	63.4 ± 3.6	0.027	NS	0.022	NS

Not significant (NS) = *p* value 0.05; K–W = Kruskal–Wallis; Mc = million cycles.
